# Liver Resection for Metastatic Colon Carcinoma

**DOI:** 10.1155/1989/40169

**Published:** 1989

**Authors:** S. Bengmark

**Affiliations:** Department of Surgery Lund University Lund 221 85 Sweden


*HPB Surgery* 1989, Vol. 1, pp. 165–170
Reprints available directly from the publisher
Photocopying permitted by license only

© 1989 Harwood Academic Publishers GmbH
Printed in Great Britain

### HPB INTERNATIONAL

**EDITORIAL & ABSTRACTING SERVICE**

JOHN TERBLANCHE, EDITOR
Department of Surgery,
Medical School · Observatory 7925
Cape Town · South Africa
Telephone: Department Office: 47-1250
Hospital Connection: 47-3311
Telex: 5-22208
Tel. Add: ALUMNI CAPETOWN
Telefax: (021) 47-8955

### LIVER RESECTION FOR METASTATIC COLON CARCINOMA

*Registry of Hepatic Metasases (Kevin S. Hughes, et al) (1988) Resection of the liver for colorectal carcinoma metastases: a multi-institutional study of indications for resection. Surgery, 103, 278–288.*

ABSTRACT

**In an investigation of the indications for hepatic resection in the treatment of colorectal carcinoma metastases, the records of 859 patients who had undergone this procedure were reviewed. This patient group, from 24 institutions, was found to have a 5-year actuarial survival of 33% and a 5-year actuarial disease-free survival of 21%. The only factors that might by themselves be considered contraindications to hepatic resection are the presence of positive hepatic nodes, the presence of resectable extrahepatic metastases, or the presence of four or more metastases. Other factors that had a negative effect on long-term survival were margins of resection on the liver metastases less than or equal to 1 cm (S [5-year actuarial survival] = 23%), the presence of positive mesenteric nodes in the primary tumor specimen (S = 23%), and a disease-free interval of less than 1 year (S = 24%). The effect of any one of these factors was not great enough to contraindicate resection. However, combinations of prognostic factors must be considered before resection is recommended. The overall 5-year survival rate for this large series has been very satisfying. Decision making in the future must take into account such factors as numbers of metastases, extrahepatic involvement, and stage of the primary tumor.**

### PAPER DISCUSSION

**Keywords:** Hepatic metastases; Liver resection; Colon carcinoma

I applaud the HPB world. It is not often that 24 leading centers from around the world cooperate by amalgamating their data to obtain a better understanding of a difficult medical problem. This group collected and studied 859 patients who had undergone curative hepatic resection for colorectal carcinoma metastases. Unfortunately their analysis extends back as far as 1948. As the field has developed so rapidly in the past 20 years it would have been better to restrict their material and concentrate on patients treated after 1965. Had they involved even more centers it may have been possible to study the data for each decade separately. As many as 64% of their patients had one single tumour resected. Their finding of a 5-year actuarial survival of 33% and a 5-year actuarial disease-free survival of 21% is similar to that reported previously by individual centers, including our own. The present study supports the conclusions from previous investigations which highlighted the negative influence on survival when there were positive hepatic nodes at the time of operation, when resectable extrahepatic metastases were present and when there were four or more metastic deposits in the liver. It also confirmed the negative effect on long-term survival when the resection margin was less than or equal to 1 cm (5-year survival 23%), the presence of positive mesenteric nodes in the original specimen for the resection of the primary tumour (5-year survival 23%) and when a disease-free interval of less than one year existed between the resection of the primary colorectal tumour and the hepatic resection for metastases (5-year survival 24%).

The analysis of the large number of patients with solitary metastases (509 patients) is of special interest. The 5-year survival was 37% in this group and the disease-free survival 25%. Note also that patients with tumours less than 4 cm fared as well with a wedge resection as when a formal anatomical liver resection was performed. However, patients with solitary metastases larger than 4 cm fared better with an anatomical liver resection. Note that their patients having anatomical resections included less Duke's C patients (51%) and less synchronous metastases (37%) than those patients who underwent a wedge resection (67% Duke's C and 57% synchronous). In my opinion it is important that attempts be made in the future to further analyse this specific group of tumours. When the present material is compared with the Lund series from 1986[1] it is interesting that both series have 36% multiple tumours. However, solitary lesions were 64% in the present study and only 49% in the Lund material. Our study contained a third group with solitary tumours with multiple small satellite tumours in the surrounding liver. The present study does not indicate where these cases are included in their data. Our study showed that these patients accounted for as many as 15% of the patients and had a great influence on survival with a 0% 3 and 5-year survival. In future studies this subset should be identified and reported separately.

It has been calculated that only some 10% of patients who are suitable for hepatic resection for colorectal metastases are referred to a surgical institution capable of undertaking the procedure because of prejudices based on three common misconceptions. These are: that hepatic metastases are fatal regardless of treatment; heptatic resection is only effective for solitary metastases; hepatic resections are accompanied by a high morbidity and a high mortality. None of these misconceptions are true today. The results of the study being reviewed emphasises the importance of such patients being referred to major centers.

In my opinion the morbidity rates can be improved significantly even though most of the complications were minor. Operative mortality should be below 5% in expert hands. When one notes that 30% of the patients with solitary tumours and as many as 18% of the patients with multiple tumours survived 5 years this provides convincing evidence favouring liver resection. Note that the resection of colorectal metastases in the liver is probably more justified than surgery for many other types of malignant lesions including primary surgery for gastric or pancreatic carcinoma. I hope that an awareness of these results will change the referral pattern in many areas of the world.

S. Bengmark
Dept of Surgery
Lund University
221 85, Lund, Sweden

### REFERENCE

1. Ekberg, H., Tranberg, K-G., Andersson, R., Lundstedt, C., Hagerstrand, I., Ranstamn, J. and Bengmark, S. (1986) Determinants of survival in liver resection of colorectal secondaries. *British Journal of Surgery*, **73**, 727–731.

### STENTING IN OBSTRUCTIVE JAUNDICE: ERCP VS PTC — NO FINAL ANSWER

*A.G. Speer, P.B. Cotton, R.C.G. Russel, R.R. Mason, A.R.W. Hatfield, J.W.C. Leung, K.D. Macrae, J. Houghton, C.A. Lennon. (1987) Randomised trial of endoscopic versus percutaneous stent insertion in malignant obstructive jaundice. Lancet ii, 57–62.*

### ABSTRACT

**Patients with biliary obstruction due to malignant disease, and judged unfit for open operation, were randomised to have a biliary stent inserted either endoscopically via the papilla of Vater or percutaneously. Analysis after 75 patients had been entered showed that the endoscopic method had a significantly higher success rate for relief of jaundice (81% versus 61%, p = 0.017) and a significantly lower 30-day mortality (15% versus 33%, p = 0.016). The higher mortality after percutaneous stents was due to complications associated with liver puncture (haemorrhage and bile leaks). When stenting is indicated in elderly and frail patients the endoscopic method should be tried first.**

